# Synergistic effects of HMG‐CoA reductase inhibitor and angiotensin II receptor blocker on load‐induced heart failure

**DOI:** 10.1002/2211-5463.12416

**Published:** 2018-04-10

**Authors:** Yusuke Ito, Yasuhiro Maejima, Natsuko Tamura, Yuka Shiheido‐Watanabe, Masanori Konishi, Takashi Ashikaga, Kenzo Hirao, Mitsuaki Isobe

**Affiliations:** ^1^ Department of Cardiovascular Medicine Tokyo Medical and Dental University Japan; ^2^ Department of Cardiology Sakakibara Heart Institute Tokyo Japan

**Keywords:** ARB, heart failure, mitophagy, pleiotropic effect, Rho kinase, statin

## Abstract

5‐Hydroxy‐3‐methylglutaryl‐CoA reductase inhibitors (statins) have beneficial effects in patients with heart failure (HF), regardless of serum cholesterol levels. However, their synergic effects with angiotensin II receptor blocker (ARB) remain to be established. We assessed the existence and potential underlying mechanisms of the effects of combined ARB [losartan (LOS)] and statin [simvastatin (SIM)] on cardiac function in rats and mice with load‐induced HF. Salt‐loaded Dahl salt‐sensitive (DS) rats were treated with vehicle, LOS, SIM, or LOS + SIM for 8 weeks. To mimic load‐induced HF *in vitro*, cultured neonatal rat cardiomyocytes (NRCM) were cyclically stretched. We also investigated the effect of LOS + SIM on pressure overload‐induced HF using mice with transverse aortic constriction (TAC). LOS + SIM improved left ventricular (LV) function and reduced LV hypertrophy more than the monotherapies in both salt‐loaded DS rats and TAC‐operated mice. LV‐tissue increases in Rho kinase and matrix metalloproteinase‐9 activity were decreased to a greater extent by LOS + SIM than by LOS and SIM monotherapies. Plasma levels of Exp‐3174, a LOS metabolite, were higher in LOS + SIM‐treated DS rats than in LOS‐treated rats. Stretch‐induced hypertrophy of NRCM pretreated with SIM + Exp‐3174 was significantly attenuated from that with LOS, Exp‐3174, SIM, or LOS + SIM. SIM administration significantly enhanced mitophagy in mouse hearts after TAC. However, LOS + SIM reduced mitophagy, and the salutary effect of SIM in mouse hearts after TAC was abolished in AT1R^−/−^ mice. In conclusion, LOS and SIM have beneficial myocardial effects on load‐induced HF via differential pleiotropic effects. Thus, combination therapy of these drugs thus has potential as a therapeutic strategy for HF.

**Abbreviations**
ARB: angiotensin II receptor blockers
AT1R: angiotensin II receptor type I
BNP: brain natriuretic peptide
BP: blood pressure
DCM: dilated cardiomyopathy
DMEM: Dulbecco's modified Eagle's medium
DS: Dahl salt‐sensitive
ELISA: enzyme‐linked immunosorbent assay
LC3: microtubule‐associated protein light‐chain 3
LOS: losartan
LVFS: left ventricular fractional shortening
LV: left ventricle
MMP‐9: matrix metalloproteinase‐9
PO: pressure overload
qRT–PCR: quantitative reverse transcription polymerase chain reaction
RAAS: renin–angiotensin–aldosterone system
RCT: randomized clinical trial
SIM: simvastatin
Statin: 5‐hydroxy‐3‐methylglutaryl (HMG)‐CoA reductase inhibitors
TAC: transverse aortic constriction
TCA: trichloroacetic acid

Increasing lines of evidence suggest that 5‐hydroxy‐3‐methylglutaryl‐CoA reductase inhibitors (statins), therapeutic agents for hypercholesterolemia, have functions in addition to those for which the statins were originally developed, called ‘pleiotropic effects’. Specifically, statins possess various effects that are independent of low‐density lipoprotein‐cholesterol lowering activity, such as anti‐inflammatory and antithrombotic activities 1. For instance, it is known that statins have a salutary effect on blood pressure (BP) by suppressing oxidative stress, improving endothelial‐mediated vasodilation, and downregulating angiotensin II type 1a receptor (AT1R) 2. Moreover, previous investigations have suggested that pleiotropic effects would exert beneficial effects on various cardiovascular diseases, including atherosclerosis and heart failure caused by ischemic heart disease and/or idiopathic dilated cardiomyopathy (DCM) 3, 4. The first report demonstrating the effectiveness of statins against heart failure was a subanalysis of the Scandinavian Simvastatin Survival Study, a large randomized, placebo‐controlled clinical trial of simvastatin (SIM) 5. The results of this randomized clinical trial (RCT) revealed that the prognosis for chronic heart failure treated with statins is more favorable than that of chronic heart failure without statin treatment. Since then, vast numbers of studies, including meta‐analyses, have shown that statins reduce the risk of mortality and hospitalization in patients with heart failure 6, 7, 8. A previous study demonstrated that the left ventricular (LV) function of DCM patients improved markedly for those undergoing SIM therapy in comparison with that of patients not undergoing SIM therapy 9. As the plasma concentrations of inflammatory cytokines were significantly lower in patients undergoing SIM therapy, the authors of that report speculated that statins might alleviate heart failure by suppressing inflammation. Another study demonstrated that SIM activates mitophagy, a selective form of autophagy targeting mitochondria, thereby attenuating ischemia/reperfusion injury in mouse hearts 10. However, the protective mechanism of statins against cardiac damage remains to be clarified.

Exacerbation of heart failure is closely related to excessive activation of the renin–angiotensin–aldosterone system (RAAS) 11. Several RCTs have demonstrated that angiotensin II receptor blockers (ARBs), drugs that potently hinder the RAAS system, improve the prognosis and symptoms of patients with heart failure 12, 13, 14. Furthermore, a line of investigations has revealed that ARBs not only block the RAAS system but also have pleiotropic effects, such as anti‐inflammatory and antifibrotic effects 15, 16.

Collectively, these findings suggest that the mechanisms of action of statins and ARBs against heart failure partially overlap and that combining these drugs can lead to more effective and safer therapies in patients with heart failure. Indeed, we have shown previously in the RCT, *HF‐COSTAR Trial,* that combined statin and ARB therapy significantly improve both symptoms and LV function over time in patients with heart failure 17. However, the beneficial synergic effects of statins and ARBs on heart failure are yet to be comprehensively understood.

In the current study, we investigated the effects of co‐administration of statin and ARB on load‐induced heart failure using salt‐loaded Dahl salt‐sensitive rats (DS rats), a model of hypertension‐induced heart failure, and mice subjected to transverse aortic constriction (TAC) surgery, a model of acute pressure overload (PO)‐induced heart failure. In addition, we elucidated a potential mechanism underlying the synergistic action of the statin and ARB.

## Materials and methods

### Experimental animals

Eight‐week‐old male DS rats (Japan SLC, Shizuoka, Japan), 10‐ to 12‐week‐old male C57BL/6J wild‐type (WT) mice (CLEA Japan, Inc., Tokyo, Japan), and AT1R knockout (AT1R^−/−^) mice (#002682; Jackson Laboratory) were used. These animals were housed in a pathogen‐free animal care facility under standard laboratory conditions (27 °C, 40–60% humidity, a 12‐h light/12‐h dark cycle) and allowed full access to standard rodent chow (CLEA Japan Inc.) and fresh water. All animal care and experimental procedures were approved by the Tokyo Medical and Dental University Guide for the Care and Use of Laboratory Animals (Permit Number: A2017‐291A) and by the Guide for the Care and Use of Laboratory Animals published by the US National Institutes of Health.

### *In vivo* experiments with DS rats

**Figure 1:**
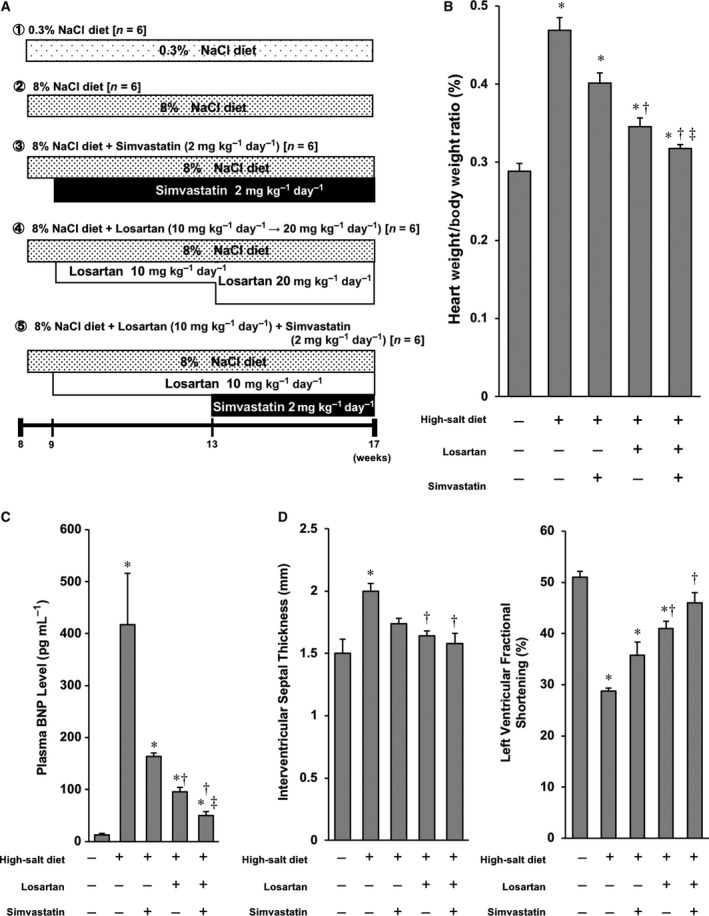
Effect of SIM and LOS on DS rats fed a high‐salt diet: (A) Protocol of *in vivo* experiments using high‐salt‐diet DS rats. (B) Bar graph of quantitative analysis of heart weight/body weight ratios. All plotted values are means ± SEM (*N* = 6). *P < 0.05 vs. control, ^†^ *P* < 0.05 vs. high‐salt‐diet DS rats with no treatment, ^‡^ *P* < 0.05 vs. high‐salt‐diet DS rats treated with LOS. (C) Bar graph of quantitative analysis of plasma BNP levels. Heart weight/body weight ratios and plasma BNP levels were significantly lower in the LOS + SIM treatment group than in the other groups. All plotted values are means ± SEM (*N* = 6). *P < 0.05 vs. control, ^†^ *P* < 0.05 vs. high‐salt‐diet DS rats with no treatment, ^‡^ *P* < 0.05 vs. high‐salt‐diet DS rats treated with LOS. (D) Left: Bar graph of quantitative analysis of interventricular septal thickness. Right: Bar graph of quantitative analysis of LVFS. All plotted values are means ± SEM (*N* = 6). *P < 0.05 vs. control, ^†^ *P* < 0.05 vs. high‐salt‐diet DS rats with no treatment.

DS rats are a well‐described animal model that is used to assess the effect of pharmacologic treatments on heart failure 18. At 8 weeks of age, the diet of DS rats was switched from a 0.3% NaCl (low‐salt) to an 8% NaCl (high‐salt) diet. Control DS rats were fed a 0.3% NaCl diet throughout the study. We performed animal experiments to compare the effects of losartan (LOS) (MERCK & Co., Inc., Kenilworth, NJ, USA), SIM (MERCK & Co., Inc.), and these drugs in combination on DS rats fed a high‐salt diet. Nine‐week‐old DS rats, which had been fed a high‐salt diet from 8 weeks of age, were given LOS (10 mg·kg^−1^·day^−1^ for 4 weeks, followed by 20 mg·kg^−1^·day^−1^ for 4 weeks), SIM (2 mg·kg^−1^·day^−1^), or LOS (10 mg·kg^−1^·day^−1^) and SIM (2 mg·kg^−1^·day^−1^) in combination for 8 weeks. LOS and SIM were suspended in 0.5% carboxymethyl cellulose and were given to the rats by gastric gavage once a day. LOS was given to the rats in their drinking water. Oral administration of 2 mg·kg^−1^·day^−1^ SIM to rats yields a plasma SIM concentration similar to that seen in patients taking clinical doses of SIM and does not significantly alter the plasma cholesterol levels in rats 19. BP was measured periodically by tail–cuff plethysmography (BP‐98A; Softron Co., Yokohama, Japan). Nine‐, 13‐, and 17‐week‐old DS rats, given a high‐salt diet for 1, 5, and 9 weeks, respectively, were anesthetized with ether; arterial blood was collected immediately by cardiac puncture; and serum and plasma were obtained by centrifugation and stored at −80 °C until use. After 8 weeks of treatment, DS rats were anesthetized with ether, and the heart was immediately excised (Fig. 1).

### Transverse aortic constriction (TAC) surgery

Transverse aortic constriction was performed in male WT and AT1R^−/−^ mice (8–12 week old, 18–25 g) as described previously 20. Briefly, mice were anesthetized with three types of mixed anesthetic agents (medetomidine, midazolam, and butorphanol at concentrations of 0.15, 2.0, and 2.5 mg·kg^−1^, respectively). Mice were placed on the warming pad in the dorsal position, and the limbs were fixed. After the skin was disinfected with 70% ethanol, the middle of the sternum was cut down to the level of the second rib. The thymus glands were then reflected to expose the transverse aorta. A 6‐0 nylon suture ligature was tied between the right innominate and left carotid artery against a blunted 27‐gauge needle, and the needle was immediately removed. The sternal cut and skin were then closed with 4‐0 sutures, and the mice were moved to a 37 °C recovery chamber. The sham operation was identical to that of TAC, without ligation of the aorta.

### Measurement of heart size and LV function

Nine weeks after induction of the high‐salt diet in DS rats and 4 weeks post‐TAC surgery in the mice, transthoracic echocardiography was performed with an ultrasound machine (Nemio; Toshiba Co., Tokyo, Japan) and a 7.5‐MHz annular array transducer under light pentobarbital sodium anesthesia. Two‐dimensional images of the heart in the short‐axis view at the level of the papillary muscles were obtained. Left ventricular fractional shortening (LVFS) was calculated as described previously 21. The average value from three consecutive beats obtained by blinded two observers was determined.

### Measurement of fibrotic area and histological analysis

After experiments, rats and mice were euthanized with an overdose intraperitoneal injection of pentobarbital sodium. The LV was fixed with ice‐cold 4% paraformaldehyde for 16–24 h and then embedded in paraffin. Five‐micrometer‐thick sections were obtained from the LV and were stained with Mallory–Azan stain as described previously 22. The stained area was identified as the area of myocardial fibrosis; this area was measured in a blinded manner at a magnification of ×200 with imagej software (National Institutes of Health, Bethesda, MD, USA) and expressed as a percentage of the tissue area in the microscopic field. Three fields of each section were averaged, and this average was taken as the fibrotic area. Perivascular fibrosis was excluded from the fibrotic area.

### Plasma and serum analyses

The plasma brain natriuretic peptide (BNP) concentration was determined by ELISA (AssayMax^®^ Rat BNP‐32 ELISA kit; Assaypro LLC, St Charles, MO, USA) according to the manufacturer's instructions. The serum total cholesterol and triglyceride concentrations were also determined by ELISA (WAKO Co., Tokyo, Japan) according to the manufacturer's instructions. The plasma LOS concentration and that of its metabolite, Exp‐3174, were analyzed by BML Laboratory (Tokyo, Japan) by means of HPLC. The sample was mixed with the mobile phase and 0.1 mol·L^−1^ TFA/acetonitrile (90/10, v/v), and 0.1 mL of this solution was analyzed on an SCL‐10A System Controller (Shimadzu Co., Kyoto, Japan) equipped with an SPD‐10A fluorescence detector (Shimadzu Co.). Chromatographic separation was carried out with a Capcell Pak C8 column and C18 column. LOS and Exp‐3174 were detected by fluorescence at excitation and emission wavelengths of 230 and 254 nm, respectively.

### *In vitro* studies of cardiomyocytes and fibroblasts from neonatal rats

Cultured cardiomyocytes from 1‐ or 2‐day‐old rats were isolated, subjected to Percoll^®^ gradient centrifugation, and plated on deformable membranes coated with collagen‐IV on Bioflex plates (Flexcell International Corp, Hillsborough, NC, USA) in MEM medium supplemented with 5% calf serum and maintained at 37 °C in humidified air with 5% CO_2_. Culture media was changed to serum‐free media 24 h prior to initiation of the experiments. Cardiac fibroblasts were obtained as described previously and cultured in Dulbecco's modified Eagle's medium supplemented with 10% FBS 23. Cells were passaged 1 : 4 at 80% confluence by trypsinization and minimal pipetting. Thirty minutes prior to stretch, LOS (200 nmol·L^−1^), SIM (10 μmol·L^−1^), or Exp‐3174 (MERCK & Co., Inc.) (200 nmol·L^−1^) was added to the culture medium. The cultured cardiomyocytes were subjected to 20% equiaxial sustained cyclic stretch (1 Hz) with an FX‐2000™ (Flexcell International Co.) strain unit equipped with loading posts 24.

### Immunoblot analyses

Immunoblot analyses were conducted as described previously 21. Briefly, to obtain protein samples, mouse LV tissues were homogenized in radioimmunoprecipitation assay (RIPA) buffer with protease inhibitor cocktail. Homogenates were centrifuged at 10 000 ***g*** for 10 min at 4 °C to remove insoluble debris. The supernatant fluid was the total cell lysate. Twenty microgram of protein was loaded per lane of a 10–20% polyacrylamide gel, separated by SDS/PAGE, and transferred to a nitrocellulose membrane. The membranes were blocked with 25% skim milk for 1 h and then incubated with 1 : 1000 diluted primary antibodies against microtubule‐associated protein light‐chain 3 (LC3) (clone 8E10, #M186‐3; MBL, Nagoya, Japan), p62 (#TA307334; ORIGENE, Rockville, MD, USA), Parkin (#ab15954; Abcam, Cambridge, UK), and β‐actin (#4967S; Cell Signaling Technology, Danvers, MA, USA) at 4 °C overnight. Membranes were washed with PBS with Tween 20 and incubated with anti‐mouse or anti‐rabbit secondary antibodies diluted 1 : 1000 with 25% skim milk for 2 h at 25 °C. Membranes were incubated with SuperSignal West Dura Extended Duration Substrate (Thermo‐Pierce, Waltham, MA, USA), and immunoreactive bands were visualized using the ChemiDoc™ MP Imaging System (Bio‐Rad Laboratories, Hercules, CA, USA). Densitometry was performed using Photoshop software.

### Gelatin zymography

For *in vivo* experiments, LV tissue was obtained 8 weeks after treatment, washed with cold PBS, and snap‐frozen in liquid nitrogen. For extraction, tissues were minced into 1‐mm^3^ pieces and incubated with 0.5% Triton X‐100 in PBS containing 0.01% sodium azide. The samples were then centrifuged, and the supernatants were collected. The matrix metalloproteinase‐9 (MMP‐9) activity of cultured cell extracts (500 ng protein) and tissue extracts (100 μg protein) was measured by in‐gel zymography with gelatin (1 mg·mL^−1^, type A from porcine skin; Sigma‐Aldrich Co., St. Louis, MO, USA) as the substrate, as described previously 23. Enzyme activity attributed to MMP‐9 was visualized as clear bands against a blue background. Recombinant human MMP‐9 (Biomol, Plymouth Meeting, PA, USA) was included in the gels as a standard. MMP‐9 activity was quantified with an imaging densitometer.

### Real‐time RT–PCR analyses

Total RNA was extracted from neonatal rat ventricular cardiomyocytes with TRIsure^®^ reagent (Nippon Genetics Co., Tokyo, Japan) and subjected to quantitative RT–PCR analysis with primers and TaqMan^®^ probes specific for rat cDNA encoding MMP‐9 (Assay ID: Rn00579162_m1) (Applied Biosystems, Foster City, CA, USA). TaqMan^®^ rodent GAPDH control reagents (VIC™ probe) were used for the detection of GAPDH mRNA as an internal standard.

### [^3^H]‐leucine incorporation

Protein synthesis was evaluated by incorporation of [^3^H]‐leucine into cultured cardiomyocytes as described previously 25. After pretreatment with or without LOS (200 nmol·L^−1^), SIM (10 μmol·L^−1^), or Exp‐3174 (200 nmol·L^−1^), cultured cardiomyocytes were subjected to 20% equiaxial sustained cyclic stretch for 20 h. L‐[3,4,5‐^3^H(N)]‐leucine (18.5 kBq; PerkinElmer Co., Waltham, MA, USA) was added, and the cultured cardiomyocytes were stretched for another 4 h. Cultured cardiomyocytes were then incubated with 5% trichloroacetic acid (TCA), and the radioactivity of an aliquot of TCA‐insoluble material was determined with a liquid scintillation counter (Model 460CD; Packard Instrument Co., Meriden, CT, USA).

### Rho kinase assays

A pull‐down assay to measure Rho activity was performed according to the manufacturer's protocol (Rho Activation Assay Kit; Cytoskeleton, Inc., Denver, CO, USA). Rho kinase activity was measured in extracts of both LV tissue and cultured cardiomyocytes. Lysates were preincubated with 100 μm nonhydrolyzable GTPγS (positive Rho activation) or 100 μm GDP (negative Rho activation) prior to precipitation with the Rhotekin GTP‐Rho binding domain (Cytoskeleton, Inc.).

### Transmission electron microscopy

Mouse heart specimens, which were harvested immediately after the mice were euthanized on day 7 after TAC surgery, were fixed in 2.5% glutaraldehyde in 0.1 m phosphate buffer for 2 h. The samples were washed with 0.1 m phosphate buffer, postfixed in 1% osmium tetroxide in the same phosphate buffer for 2 h, dehydrated in a graded series of ethanol washes, and embedded in Epon 812 (TAAB, Aldermaston, UK). Semi‐thin sections were sliced at 1 μm and stained with toluidine blue. Ultrathin 90‐nm sections were collected on copper grids, double‐stained with uranyl acetate and lead citrate, and then observed using transmission electron microscopy (H‐7011; Hitachi, Tokyo, Japan).

### Statistics

All statistical analyses were conducted with ibm spss statistics 22 software (SPSS Japan Institute, Tokyo, Japan). All statistical data are presented as means ± SEM. Multiple comparisons were conducted using one‐way anova, followed by Tukey's *post hoc* test for homoscedastic data or Games–Howell *post hoc* test for heteroscedastic data. In all cases, results were considered statistically significant at a *P* < 0.05.

## Results

### Co‐administration of SIM and LOS alleviates cardiac dysfunction and heart failure in DS rats fed high‐salt diet

To address the effects of LOS, SIM and their combination on load‐induced heart failure, LOS (10 mg·kg^−1^·day^−1^ for 4 weeks and then 20 mg·kg^−1^·day^−1^ for 4 weeks), SIM (2 mg·kg^−1^·day^−1^), or LOS (10 mg·kg^−1^·day^−1^) and SIM (2 mg·kg^−1^·day^−1^) in combination were administered for 8 weeks (Fig. 1A). In the DS rats, the high‐salt diet produced systemic hypertension. LOS, SIM, and LOS + SIM all reduced BP significantly throughout the treatment period compared to the level in untreated rats. However, there was no significant difference between drugs in the level of BP reduction. After 8 weeks of treatment, there was no significant difference in plasma cholesterol or triglyceride levels between groups of DS rats (Table 1). Next, we measured heart weight/body weight and plasma BNP levels in the rats at 17 weeks of age (Fig. 1B,C). Both the heart weight/body weight and plasma BNP levels of high‐salt‐diet DS rats were significantly higher than those of untreated DS rats. Treatment with LOS, but not SIM, significantly reduced these elevated heart weight/body weight and plasma BNP levels. LOS and SIM in combination also reduced these two indicators of heart failure. Echocardiographic examinations showed that LV wall thickness increased significantly in the high‐salt‐diet DS rats. Treatment with LOS, but not SIM, significantly decreased LV wall thickness, and LOS and SIM in combination decreased LV wall thickness more than LOS monotherapy did in high‐salt‐diet DS rats. High‐salt‐diet DS rats showed the decrease in LVFS. Although SIM alone did not significantly improve systolic function as determined by LVFS, LOS alone and LOS and SIM in combination significantly improved systolic LV function (Fig. 1D). Furthermore, the LVFS with LOS + SIM was significantly greater than that with LOS alone. These results suggest that co‐administration of SIM and LOS effectively alleviates hypertension‐induced cardiac dysfunction and heart failure in DS rats.

**Table 1:** BP and circulating lipid levels in DS rats

	Control (*N* = 6)	No‐treatment (*N* = 6)	SIM (*N* = 6)	LOS (*N* = 6)	SIM + LOS (*N* = 6)
Systolic BP (mmHg)	113 ± 3.2	175.6 ± 10.4*	141.4 ± 4.0*	126 ± 3.0†	122 ± 4.0†
Diastolic BP (mmHg)	74 ± 6.9	125 ± 2.9*	105 ± 4.6*	93 ± 3.2*, †	90 ± 2.1*, †
Plasma cholesterol (mg·dL^−1^)	94 ± 22.3	79 ± 19.6	85 ± 16.1	84 ± 23.2	84 ± 14
Plasma triglyceride (mg·dL^−1^)	46 ± 15.1	44 ± 11.1	43 ± 3.3	38 ± 6.2	43 ± 8.7

Values are mean ± SEM, *P < 0.05 vs. control. ^†^ *P* < 0.05 vs. high‐salt‐diet DS rats without treatment.

### Co‐administration of SIM and LOS suppresses cardiac hypertrophy and myocardial fibrosis in DS rats fed high‐salt diet

**Figure 2:**
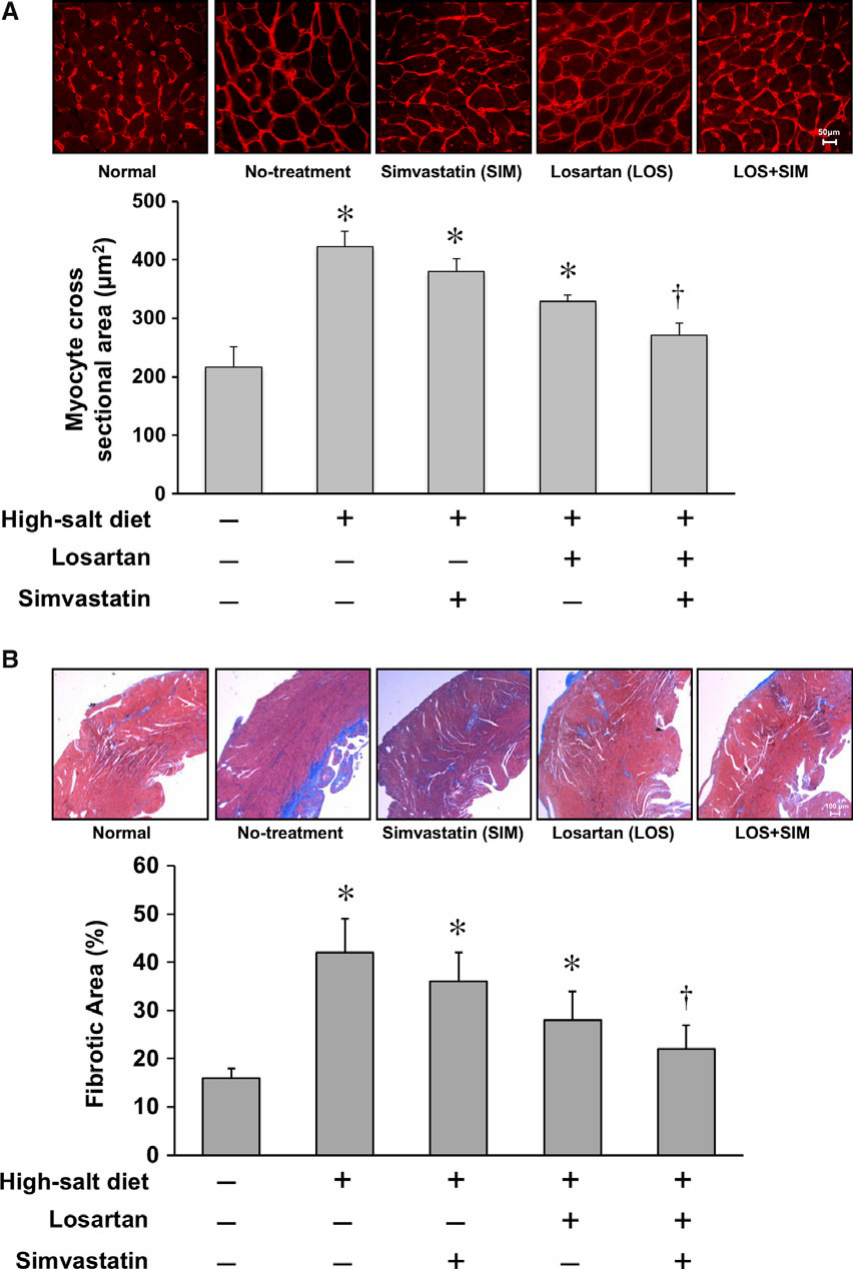
Effect of SIM and LOS on cardiomyocyte diameter and cardiac fibrosis in DS rats fed a high‐salt diet: (A) Upper: Representative photomicrographs of wheat germ agglutinin (WGA)‐stained left ventricle (LV) sections of DS rats. Lower: Cross‐sectional areas of LV cardiomyocytes are plotted as means ± SEM. *N* = 3 per group. *P < 0.05 vs. control, ^†^ *P* < 0.05 vs. high‐salt‐diet DS rats with no treatment. (B) Upper: Representative photomicrographs of Mallory–Azan‐stained LV sections of DS rats. Lower: Percentage areas of fibrosis in the LV walls are plotted as means ± SEM. *N* = 3 per group. *P < 0.05 vs. control, ^†^ *P* < 0.05 vs. high‐salt‐diet DS rats with no treatment.

We measured cardiomyocyte cross‐sectional area in wheat germ agglutinin‐stained tissue sections. The cardiomyocyte cross‐sectional area in high‐salt‐diet DS rats was significantly larger than that in normal‐diet DS rats (Fig. 2A). Although neither LOS nor SIM alone significantly decreased the size of cardiomyocytes, LOS and SIM in combination markedly decreased the cardiomyocyte cross‐sectional area. We also evaluated LV fibrosis in Mallory–Azan‐stained sections and found that the LV fibrotic area was significantly higher in high‐salt‐diet DS rats than in normal‐diet DS rats. Although neither LOS nor SIM alone decreased fibrosis, LOS and SIM in combination markedly suppressed the fibrosis of LV (Fig. 2B). These results suggest that co‐administration of SIM and LOS effectively inhibits hypertension‐induced cardiomyocyte hypertrophy and myocardial fibrosis in DS rats.

### Co‐administration of SIM and LOS attenuates the activities of both Rho kinase and MMP‐9 in LV tissue of DS rats fed high‐salt diet

**Figure 3:**
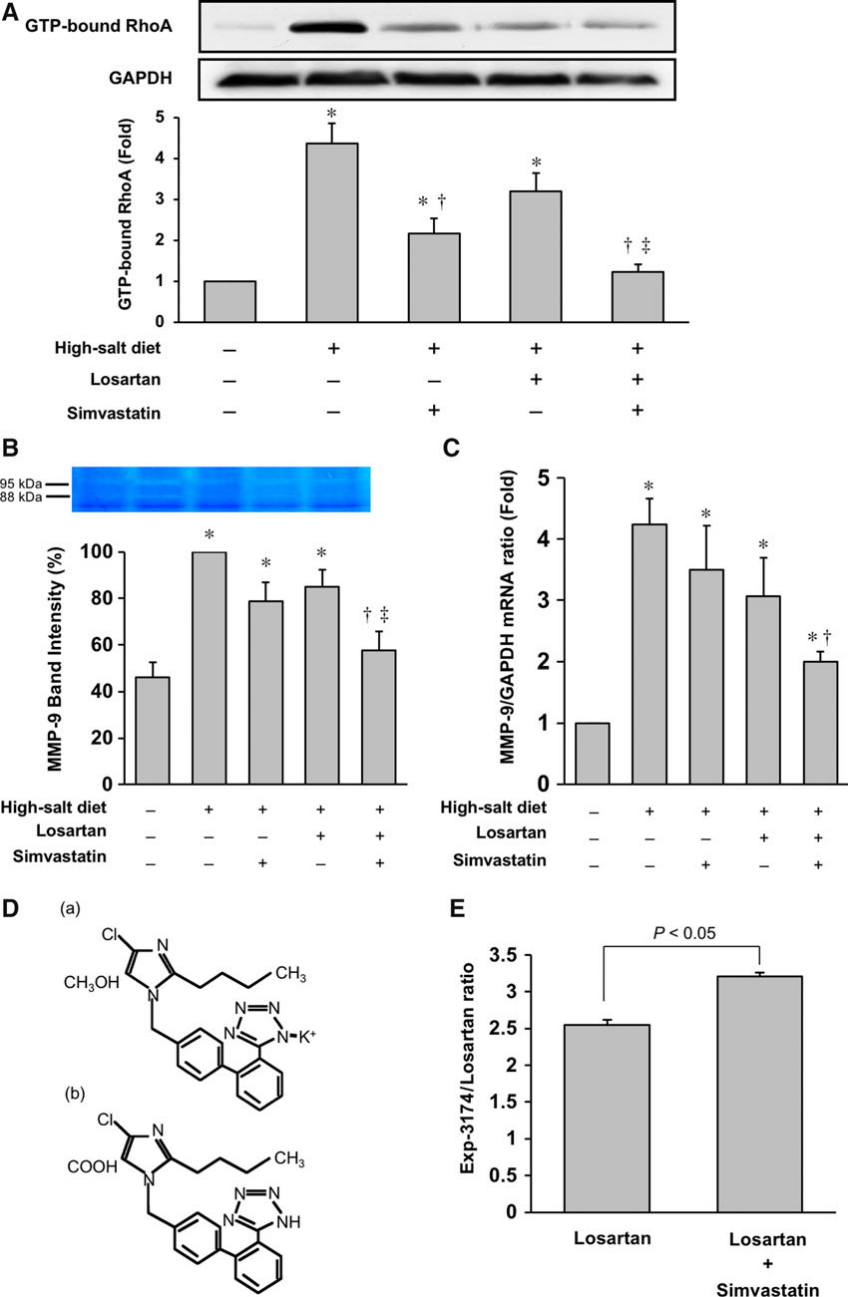
Potential mechanisms of SIM and LOS activity on cardiac remodeling in DS rats fed a high‐salt diet: (A) Evaluation of Rho kinase activity in myocardium of DS rats. Rho kinase activity was analyzed by pull‐down assay with the Rhotekin GTP‐Rho‐binding domain. Whole‐cell extracts were prepared and analyzed. A representative immunoblot is shown above a bar graph of Rho kinase activity evaluated by band density. (B) Upper: Representative image of in‐gel gelatin zymography for MMP‐9 from the myocardium of DS rats. Lower: Bar graph of quantitative analysis of MMP‐9 activity evaluated by band intensity. (C) Bar graph of quantitative analysis of MMP‐9 mRNA expression in the myocardium of DS rats. All plotted values are means ± SEM (*N* = 3). *P < 0.05 vs. control, ^†^ *P* < 0.05 vs. high‐salt‐diet DS rats with no treatment, ^‡^ *P* < 0.05 vs. high‐salt‐diet DS rats treated with LOS. (D) (a) Structure of LOS (MK‐954), a competitive antagonist of the AT1 receptor. (b) Structure of Exp‐3174, a metabolite of LOS and inverse agonist of the AT1 receptor. (E) Ratio of the plasma Exp‐3174/LOS concentration in DS rats. Plotted values are means ± SEM (*N* = 9). The plasma Exp‐3174 level was higher with LOS and SIM cotreatment than with LOS treatment alone.

We next examined the mechanism underlying the effect of LOS and SIM in combination on high‐salt‐diet‐induced heart failure in DS rats. Pull‐down assays demonstrated that Rho kinase activity of LV tissue in DS rats was significantly increased by the high‐salt diet. LOS and SIM in combination significantly attenuated the Rho kinase activity in LV tissue; this attenuation was greater than that by LOS or SIM alone (Fig. 3A). The gelatinase activity of MMP‐9 (95/88 kDa) in the high‐salt‐diet DS rats was significantly higher than that in the low‐salt‐diet DS rats, as shown by in‐gel gelatin zymography. This increased activity was significantly lower in the LOS group and in the SIM group than that in the nontreatment group. LOS and SIM in combination further reduced MMP‐9 activity (Fig. 3B). MMP‐9 mRNA expression in the high‐salt‐diet DS rats was also significantly higher than that in the control DS rats. This upregulation was significantly lower in the LOS groups and in the SIM group than in the nontreatment group. LOS and SIM in combination reduced MMP‐9 mRNA expression more than did either agent alone (Fig. 3C). These results suggest that co‐administration of SIM and LOS attenuates the activities of both Rho kinase and MMP‐9 in the LV tissue of DS rats.

### Co‐administration of SIM and LOS increases plasma levels of Exp‐3174

Increasing lines of evidence suggest that LOS, a competitive antagonist of AT1R, is converted to Exp‐3174, an inverse agonist of AT1R, in the liver 26, 27. To elucidate whether administration of SIM affects the metabolism of LOS in DS rats, we determined the circulating levels of Exp‐3174 in DS rats treated for 8 weeks with LOS alone or LOS and SIM in combination. The plasma Exp‐3174 level was higher with combination LOS and SIM treatment than with LOS alone (Fig. 3D). Thus, co‐administration of SIM and LOS would be expected to enhance the plasma level of Exp‐3174.

### Stretch‐induced hypertrophy is suppressed by cotreatment with SIM and Exp‐3174

**Figure 4:**
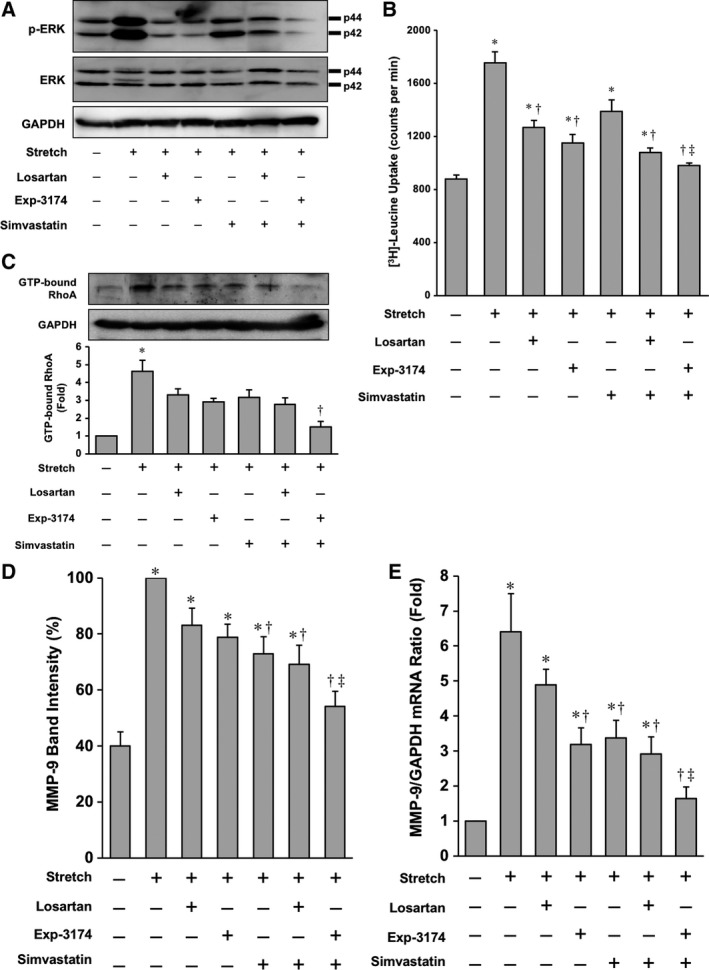
Effect of SIM, LOS, and Exp‐3174 on cultured neonatal rat cardiomyocytes and fibroblasts subjected to stretch: (A) Activation of ERK by mechanical stretch. Neonatal rat ventricular cardiomyocytes were pretreated with LOS (200 nmol·L^−1^), SIM (10 μmol·L^−1^), Exp‐3174 (200 nmol·L^−1^), LOS + SIM or Exp‐3174 + SIM and stretched by 20%. Activation of ERK was determined by p‐ERK immunoblot. Representative blots are shown. (B) Protein synthesis of neonatal rat ventricular cardiomyocytes was assessed by [^3^H]‐leucine incorporation. Values shown are means ± SEM of three dishes. Similar results were obtained in at least three independent experiments. *P < 0.05 vs. control, ^†^ *P* < 0.05 vs. stretched neonatal rat ventricular cardiomyocytes with no treatment, ^‡^ *P* < 0.05 vs. stretched neonatal rat ventricular cardiomyocytes treated with LOS. (C) Evaluation of Rho kinase activity in cardiac fibroblasts. Representative immunoblots are shown above a bar graph of quantitative analysis of Rho kinase activity evaluated by band density. (D) Bar graph of MMP‐9 activity in cardiac fibroblasts evaluated by zymography. (E) Bar graph of MMP‐9 mRNA expression in cardiac fibroblasts. Plotted values are means ± SEM (*N* = 3). *P < 0.05 vs. control, ^†^ *P* < 0.05 vs. stretched neonatal rat cardiac fibroblasts with no treatment, ^‡^ *P* < 0.05 vs. stretched neonatal rat cardiac fibroblasts treated with LOS.

We examined the effect of statin and ARB on phosphorylation of ERK in stretched cultured cardiomyocytes. As shown by immunoblot analyses, mechanical stretching of cultured cardiomyocytes by 20% activated ERKs, and this activation was significantly suppressed by pretreatment with both SIM and Exp‐3174. The inhibitory effect of ERK activation was more prominent than that by LOS, Exp‐3174, or SIM alone or by other combinations (Fig. 4A). To assess the antihypertrophic effect of both statin and ARB, stretched cultured cardiomyocytes were treated with LOS, Exp‐3174, or SIM. Pretreatment with SIM and Exp‐3174 in combination significantly inhibited the stretch‐induced increase in [^3^H]‐leucine incorporation. The inhibition was more marked than that by LOS, Exp‐3174, or SIM alone or by other combinations (Fig. 4B). These results indicate that statin and ARB in combination effectively inhibit the stretch‐induced activation of ERKs and attenuate hypertrophy in cultured cardiomyocytes.

### Cotreatment with SIM and Exp‐3174 attenuates the activities of both Rho kinase and MMP‐9 in cardiac fibroblasts

Rho kinase and MMP‐9 activities of stretched cardiac fibroblasts were evaluated under various pretreatment conditions. Rho kinase activity was upregulated by the stretching, and this activation was attenuated by pretreatment with SIM and Exp‐3174 in combination more than that by LOS, Exp‐3174, or SIM alone or by other combinations (Fig. 4C). The gelatinase activity of MMP‐9 in cardiac fibroblasts was also increased under this condition, and this increased activity was downregulated by pretreatment with SIM and Exp‐3174 in combination to a greater extent than that with LOS, Exp‐3174, or SIM alone or with other combinations (Fig. 4D). We then examined the effect of stretching of cardiac fibroblasts on MMP‐9 mRNA expression by real‐time RT–PCR. Stretch stimulation significantly increased MMP‐9 mRNA expression in cardiac fibroblasts, and pretreatment with SIM and Exp‐3174 in combination abolished the increase in MMP‐9 expression (Fig. 4E).

### Co‐administration of SIM and LOS alleviates cardiac dysfunction and histopathological changes in mouse hearts in response to PO

**Figure 5:**
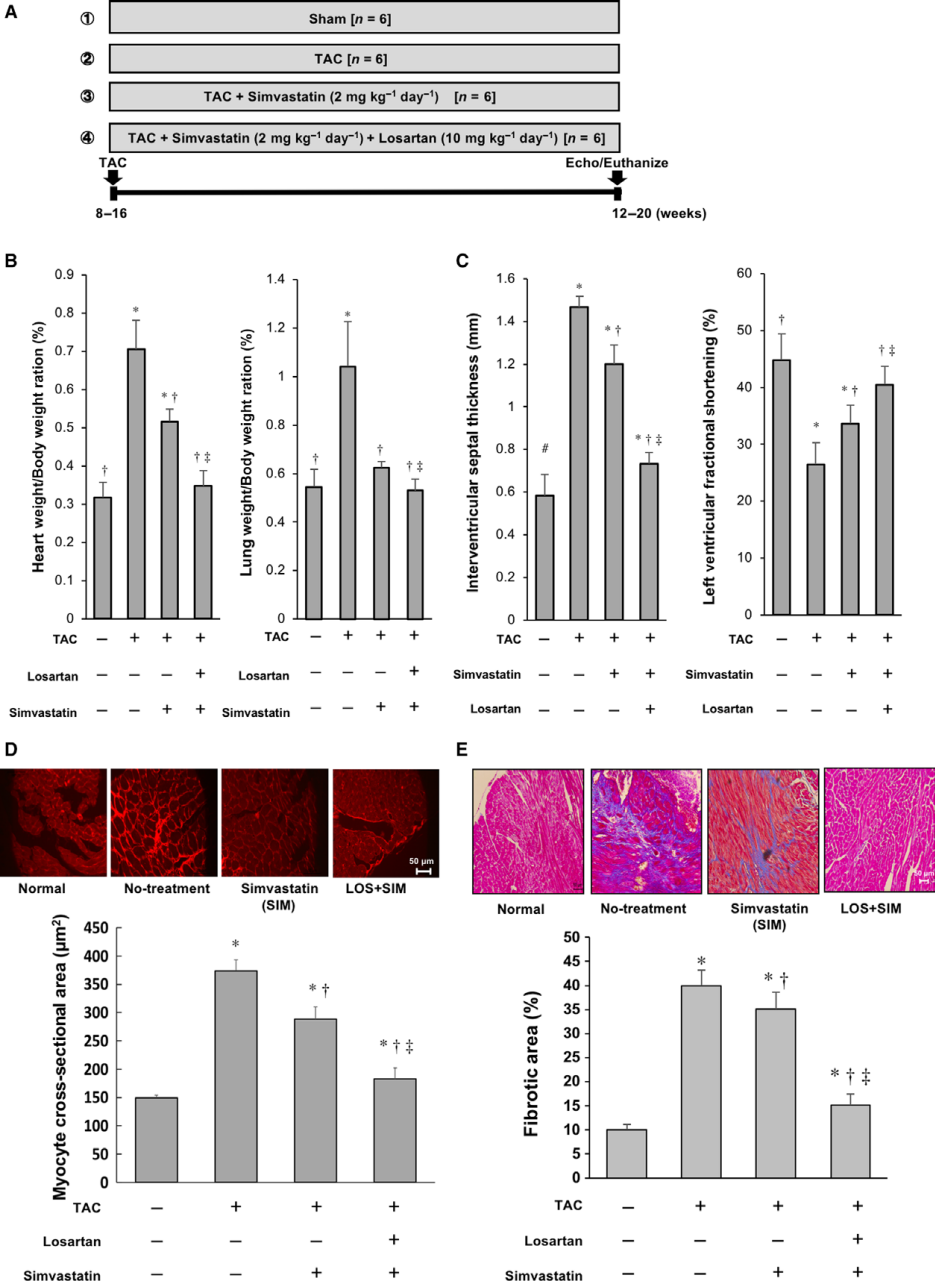
Effect of SIM and LOS on mice subjected to TAC: (A) Protocol of *in vivo* experiments using mice subjected to TAC surgery. (B) Left: Bar graph of quantitative analysis of heart weight/body weight ratio. Right: Bar graph of quantitative analysis of lung weight/body weight ratio. (C) Left: Bar graph of quantitative analysis of interventricular septal thickness. Right: Bar graph of quantitative analysis of LVFS. (D) Upper: Representative photomicrographs of wheat germ agglutinin (WGA)‐stained LV sections of mice. Lower: Quantitative plots of the LV cardiomyocyte areas are presented. (E) Upper: Representative photomicrographs of Mallory–Azan‐stained LV sections of mice. Lower: Areas of LV cardiac fibrosis are plotted. Plotted values are means ± SEM (*N* = 3). *P < 0.05 vs. sham‐operated mice, ^†^ *P* < 0.05 vs. TAC‐operated mice with no treatment, ^‡^ *P* < 0.05 vs. TAC‐operated mice with SIM.

To further confirm the effects of LOS and SIM on cardiac function and histology in load‐induced heart failure, we used a TAC mouse model (Fig. 5A). LOS treatment under PO significantly reduced peripheral BP throughout the treatment period. In contrast, SIM treatment did not affect BP during PO (Table 2). The increase in both LV and lung weights was significantly reduced by the treatment with SIM alone, and, importantly, LOS and SIM in combination additionally reduced the two weights after PO (Fig. 5B). Echocardiographic examinations demonstrated that treatment with SIM and LOS in combination, but not SIM alone, significantly reduced interventricular septal thickness and improved LVFS (Fig. 5C; Table 3). Histological analysis revealed that SIM and LOS in combination significantly decreased both cardiomyocyte cross‐sectional area and fibrotic area more than did SIM monotherapy in TAC mice (Fig. 5D,E). Collectively, these results indicate that SIM and LOS in combination improve LV morphology and function better than SIM monotherapy does in response to PO.

**Table 2:** BP in TAC‐operated mice

	Sham (*N* = 6)	TAC (*N* = 6)	TAC + SIM (*N* = 6)	TAC + SIM + LOS (*N* = 6)
Systolic BP (mmHg)	115.2 ± 1.9	96.2 ± 1.4*	96.9 ± 2.0*	88.9 ± 1.0*, †
Diastolic BP (mmHg)	54.7 ± 1.6	44.4 ± 1.0*	44.3 ± 1.0*	38.6 ± 0.8*, †

Values are means ± SEM, *N* = 6 per group. *P < 0.05 vs. Sham. ^†^ *P* < 0.05 vs. TAC mice without treatment.

**Table 3:** Echocardiography parameters of LV function in TAC‐operated mice

	Sham (*N* = 6)	TAC (*N* = 6)	TAC + SIM (*N* = 6)	TAC + SIM + LOS (*N* = 6)
EDD (mm)	3.50 ± 0.07	4.05 ± 0.56*	3.72 ± 0.13	3.30 ± 0.05†
ESD (mm)	2.23 ± 0.08	3.18 ± 0.16*	2.92 ± 0.17*	2.55 ± 0.10†
IVS (mm)	0.58 ± 0.10	1.47 ± 0.05*	1.20 ± 0.09*, †	0.73 ± 0.05*, †
PW (mm)	0.62 ± 0.03	1.35 ± 0.03*	1.17 ± 0.02*, †	0.72 ± 0.03†
FS	44.83 ± 4.62	26.5 ± 3.83*	33.67 ± 3.27*, †	40.5 ± 3.21*, †

EDD, end‐systolic dimension; ESD, end‐diastolic dimension; IVS, interventricular septum thickness; PW, posterior wall thickness; fractional shortening (FS) were measured by M‐mode echocardiography. Values are means ± SEM, *N* = 6 per group. *P < 0.05 vs. Sham. ^†^ *P* < 0.05 vs. TAC mice without treatment.

### Simvastatin alleviates load‐induced heart failure, possibly by activating mitophagy

**Figure 6:**
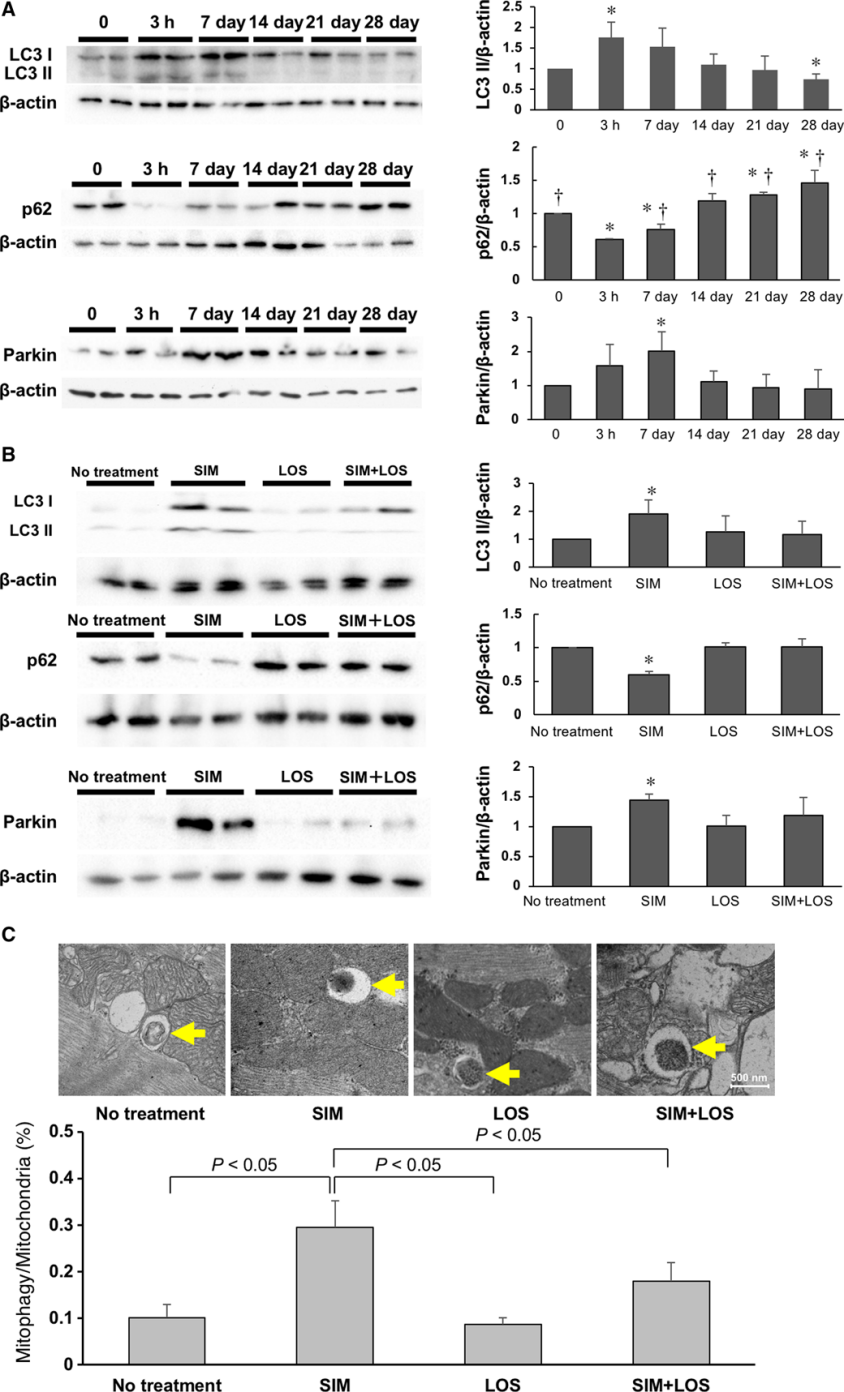
Effect of SIM and LOS on autophagy and mitophagy in mice subjected to TAC: (A) Upper: Representative immunoblots (left) and quantitative analysis (right) of whole‐heart homogenates for LC3 and β‐actin, 0, 3 h, 7 days, 14 days, 21 days, and 28 days after TAC (*N* = 3 in each group). Middle: Representative immunoblots (left) and quantitative analysis (right) of whole‐heart homogenates for p62 and β‐actin, 0, 3 h, 7 days, 14 days, 21 days, and 28 days after TAC (*N* = 3 in each group). Lower: Representative immunoblots (left) and quantitative analysis (right) of whole‐heart homogenates for Parkin and β‐actin, 0, 3 h, 7 days, 14 days, 21 days, and 28 days after TAC (*N* = 3 in each group). *P < 0.05 vs. 0 h mice (*N* = 3), ^†^ *P* < 0.05 vs. 3 h mice (*N* = 3). Data are expressed as means ± SEM. (B) Upper: Representative immunoblots (left) and quantitative analysis (right) of whole‐heart homogenates for LC3 and β‐actin 7 days after TAC (*N* = 3 in each group). Middle: Representative immunoblots (left) and quantitative analysis (right) of whole‐heart homogenates for p62 and β‐actin 7 days after TAC (*N* = 3 in each group). Lower: Representative immunoblots (left) and quantitative analysis (right) of whole‐heart homogenates for Parkin and β‐actin 7 days after TAC (*N* = 3 in each group). *P < 0.05 vs. TAC‐operated mice with no treatment. Data are expressed as means ± SEM. (C) Upper: Representative transmission electron microscopy images of mitochondria during mitochondrial autophagy in the myocardium of TAC‐operated mice. Arrows indicate mitochondria in autophagosomes. Scale bar: 500 μm. Lower: Bar graph of quantitative analysis of mitophagy / mitochondria ratio. Data are expressed as means ± SEM (*N* = 3 in each group). LC3, microtubule‐associated protein light‐chain 3.

Increasing lines of evidence suggest that mitophagy plays a protective role in the heart, including during load‐induced heart failure 28, 29. We addressed the effect of co‐administration of LOS and SIM on mitophagy in the hearts with PO. Consistent with a previous report 29, immunoblot analyses demonstrated that the level of LC3II in the myocardium, a marker of general autophagy, was significantly increased 3 h after TAC. Similarly, p62, an adaptor protein degraded by autophagy, was significantly decreased 3 h after TAC. The protein level of Parkin, the other important mitophagy‐associated protein, was significantly increased 7 days after TAC. Then, the levels of both LC3II and Parkin were significantly decreased and that of p62 was markedly increased 14 days after TAC (Fig. 6A). When administering SIM, both LC3II and Parkin significantly increased and p62 significantly decreased 7 days after TAC (Fig. 6B), as shown in a previous report 10. However, unexpectedly, co‐administration of LOS and SIM attenuated the accumulation of LC3II and Parkin and the decreasing of p62 caused by SIM administration (Fig. 6B). Ultramicroscopic examinations demonstrated that the number of mitochondria in autophagosomes was significantly increased by SIM treatment alone and that co‐administration of LOS and SIM reduced these numbers in the myocardium 7 days after TAC (Fig. 6C). These results suggest that SIM enhances activation of PO‐induced mitophagy and that AT1R‐mediated signaling is required for activation of mitophagy.

### AT1R‐mediated signaling is required for cardioprotective effect by co‐administration of SIM and LOS

**Figure 7:**
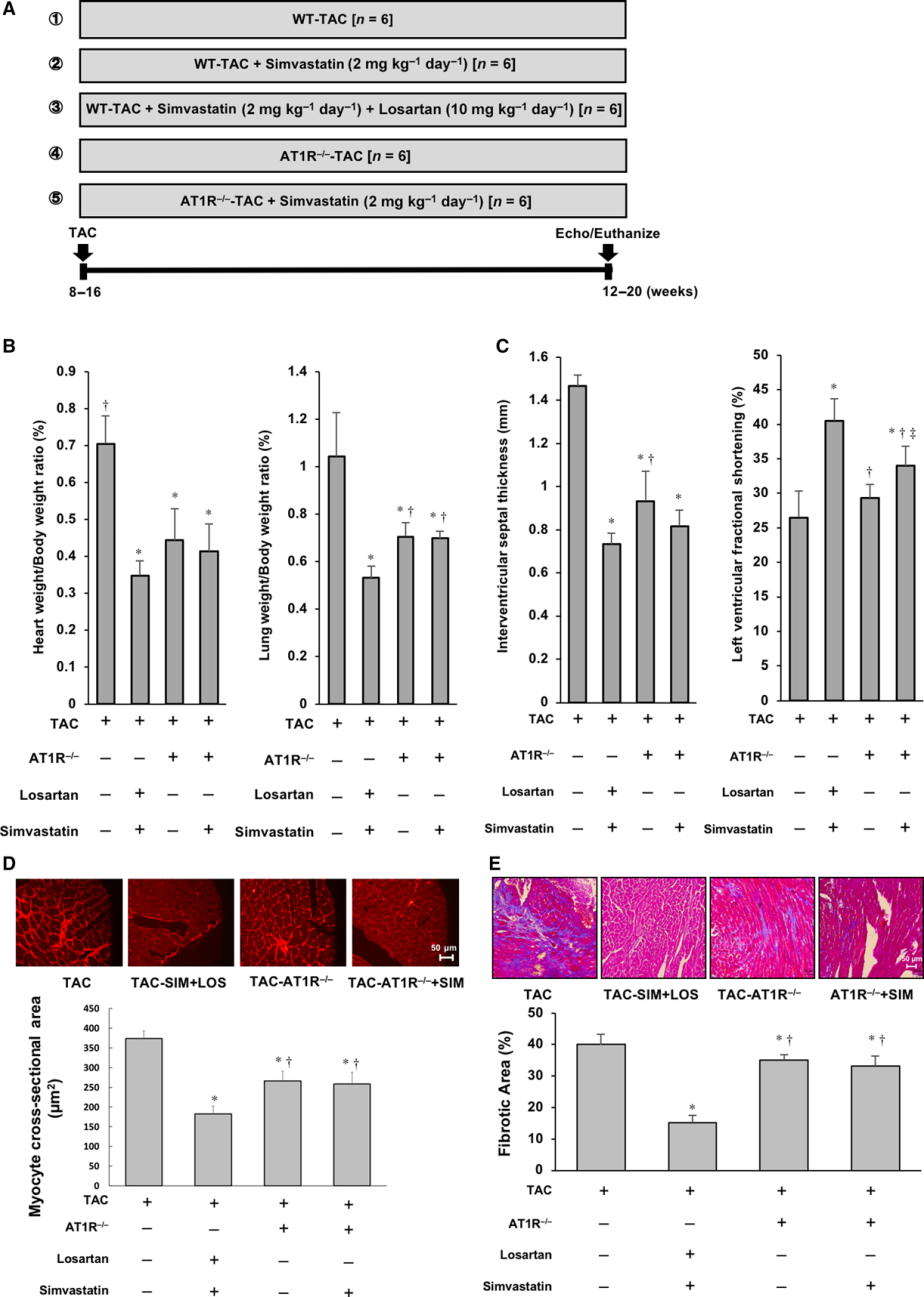
Effect of SIM and LOS on WT and AT1R^−/−^ mice subjected to TAC: (A) Protocol of *in vivo* experiments using mice subjected to TAC surgery. (B) Left: Bar graph of heart weight/body weight ratios. Right: Bar graph of lung weight/body weight ratios. (C) Left: Bar graph of quantitative analysis of interventricular septal thickness. Right: Bar graph of quantitative analysis of LVFS. (D) Upper: Representative photomicrographs of wheat germ agglutinin (WGA)‐stained LV sections of mice. Lower: LV cardiomyocyte areas are plotted. (E) Upper: Representative photomicrographs of Mallory–Azan‐stained LV sections of mice. Lower: Percentage area of cardiac fibrosis in the LV wall is plotted. Plotted values are means ± SEM (*N* = 3). *P < 0.05 vs. TAC‐operated WT mice with no treatment, ^†^ *P* < 0.05 vs. TAC‐operated WT mice with LOS + SIM, ^‡^ *P* < 0.05 vs. TAC‐operated AT1R^−/−^ mice with no treatment.

We further assessed the mechanism of cardioprotective effects by co‐administration of SIM and LOS using the TAC model in AT1R^−/−^ mice to address whether synergic effects of statin and ARB co‐administration are principally mediated through broad suppression of AT1R‐mediated signaling (Fig. 7A). Deletion of the AT1R gene and LOS administration led to significantly lower systolic and diastolic BP than that in untreated WT mice with PO (Table 4). The lung weight/body weight ratio of WT mice treated with SIM + LOS was significantly lower than that of AT1R^–/–^ mice treated with SIM alone, in response to PO (Fig. 7B). Similarly, the LV systolic function of WT mice treated with SIM + LOS was significantly greater than that of AT1R^–/–^ mice treated with SIM, in response to PO (Fig. 7C). Regarding heart weight/body weight ratio and interventricular septal thickness, there were no significant differences between WT mice treated with SIM + LOS and AT1R^−/−^ mice treated with SIM. However, these indicators tended to be lower in WT mice treated with SIM + LOS than in AT1R^−/−^ mice treated with SIM only (Fig. 7B,C; Table 5). Histopathologic examination demonstrated that both cardiomyocyte size and LV fibrotic area of WT mice treated with SIM + LOS were markedly smaller than those of AT1R^−/−^ mice treated with SIM (Fig. 7D,E). These results suggest that the salutary effects of SIM + LOS co‐administration in response to PO in the heart are mediated through the synergy of various SIM‐mediated pleiotropic effects and those of LOS‐mediated inverse agonistic activity of the AT1R, and that complete ablation of AT1R is not beneficial for load‐induced heart failure.

**Table 4:** BP in TAC‐operated mice

	WT‐TAC (*N* = 6)	WT‐TAC + SIM + LOS (*N* = 6)	AT1R^−/−^ − TAC (*N* = 6)	AT1R^−/−^ − TAC + SIM (*N* = 6)
Systolic BP (mmHg)	96.2 ± 1.4	88.9 ± 1.0*	89.5 ± 1.1*	89.5 ± 0.8*
Diastolic BP (mmHg)	44.4 ± 1.0	38.6 ± 0.8*	39.9 ± 0.6*	39.4 ± 1.2*

Values are mean ± SEM, *N* = 6 per group. *P < 0.05 vs. WT‐TAC group without treatment.

**Table 5:** Echocardiography parameters of LV function in TAC‐operated mice

	WT‐TAC (*N* = 6)	WT‐TAC + SIM + LOS (*N* = 6)	TAC + AT1R^−/−^ (*N* = 6)	TAC + AT1R^−/−^ + SIM (*N* = 6)
EDD (mm)	4.05 ± 0.56	3.30 ± 0.05†	3.77 ± 0.08	3.51 ± 0.04†
ESD (mm)	3.18 ± 0.16	2.55 ± 0.10†	2.62 ± 0.15*	2.73 ± 0.07†
IVS (mm)	1.47 ± 0.05	0.73 ± 0.05*^†^	0.93 ± 0.14*^†^	0.73 ± 0.05*
PW (mm)	1.35 ± 0.03	0.72 ± 0.03†	1.17 ± 0.02*^†^	0.72 ± 0.03†
FS	26.5 ± 3.83	40.5 ± 3.21*	29.33 ± 1.97*^†^	34 ± 2.83*^†^, ‡

EDD, end‐systolic dimension; ESD, end‐diastolic dimension; IVS, interventricular septum thickness; PW, posterior wall thickness; fractional shortening (FS) were measured by M‐mode echocardiography. Values are means ± SEM, *N* = 6 per group. *P < 0.05 vs. TAC‐operated WT mice with no treatment, ^†^ *P* < 0.05 vs. TAC‐operated WT mice with LOS + SIM, ^‡^ *P* < 0.05 vs. TAC‐operated AT1R^−/−^ mice with no treatment.

## Discussion

In the present study, LOS and SIM were co‐administered to rat and mouse models of load‐induced heart failure, and the combination therapy was shown to be significantly more effective in the treatment of heart failure than were the individual monotherapies. The beneficial pleiotropic effects of LOS and SIM against heart failure were obviously enhanced by combining the two drugs, possibly through elevating the plasma concentration of Exp‐3174, a LOS metabolite possessing greater inverse agonistic action than LOS itself. We also found that SIM‐mediated enhancement of mitophagy, which plays a beneficial role in alleviating PO‐induced cardiac dysfunction, is suppressed by the presence of LOS. However, in response to PO, the cardioprotective effects of SIM + LOS co‐administration were significantly greater than those of SIM alone in the absence of AT1R, suggesting that some type of AT1R‐mediated signaling, such as a negative signal stimulated by inverse agonists, would be essential against load‐induced heart failure.

With regard to the usefulness of combining statin and ARB in the treatment of heart failure, we first focused on mutual enhancement of the pleiotropic effects of statin and ARB. Statins exert pleiotropic effects on various organs and cells. Although various molecules are involved, it has been clarified that Rho kinase, a low‐molecular‐weight G protein, is an important molecule for regulating the pleiotropic effects of statins 30, 31, 32. We have shown that statin prevents angiotensin II‐induced cardiac hypertrophy via inhibition of cyclin D1 expression and attenuation of Rho kinase activity 25. In addition, previous investigations have demonstrated that stretching activates RhoA in cardiomyocytes and that inhibition of Rho kinase suppresses stretch‐induced cardiac hypertrophy 33, 34. Rho kinase also plays important roles in angiotensin II‐induced hypertrophic responses in cardiomyocytes 35. Increasing evidence suggests that myocardial fibrosis is critical for heart failure because fibrosis of myocardial tissues can impede normal heart function 36, 37, 38. In addition, it is known that MMP‐9 plays a central role in this pathologic process and that the activity of MMP‐9 is critically regulated by Rho kinase‐mediated pathway 39, 40, 41. Thus, we focused on the effects of statin and ARB administered in combination on Rho kinase and MMP‐9 activation. Indeed, the results of this study revealed that LOS + SIM suppresses Rho kinase and MMP‐9 activity more potently *in vivo* than do the individual monotherapies.

Basically, ARBs are classified by function as competitive antagonists or inverse agonists. Competitive antagonists hinder signal transduction by competing with angiotensin II for AT1Rs. However, inverse agonists bind to AT1Rs to generate negative signals. Consequently, competitive antagonists cannot suppress AT1R stimulation other than that by angiotensin II; thus, AT1R activation due to stretch stimulation can be suppressed only by inverse agonists 42. Therefore, an ARB possessing inverse agonist activity is more effective against heart failure, particularly heart failure caused by PO, such as hypertension or valvular heart disease. Although LOS is a competitive antagonist of AT1R, its metabolite, Exp‐3174, is a potent inverse agonist 26, 27. In addition, LOS is metabolized by cytochrome P‐450 enzymes (CYPs: CYP3A4 and CYP2C9) 43. Furthermore, SIM enhances the activity of drugs metabolized by CYPs, and the synergism may involve metabolism by these enzymes 44, 45, 46. These findings led us to hypothesize that co‐administration of SIM promotes conversion of LOS to Exp‐3174, thereby demonstrating potent cardioprotective effects. Consistent with our hypothesis, we found that the plasma concentration of Exp‐3174 after combination therapy of LOS + SIM was significantly higher than that following LOS monotherapy, and that treatment with SIM and LOS in combination significantly improved LV function more than did LOS alone in load‐induced heart failure models. In addition, a series of *in vitro* experiments of stretching both cultured cardiomyocytes and cardiac fibroblasts strongly support our hypothesis. Co‐administration of SIM and Exp‐3174 was most effective in suppressing hypertrophy of cultured cardiomyocytes. The activities of both Rho kinase and MMP‐9, key factors for promoting cardiac fibrosis 39, 40, 41, were also effectively suppressed by co‐administration of SIM and Exp‐3174. Taken together, these findings suggest that treatment with LOS and SIM in combination suppresses both cardiac hypertrophy and myocardial fibrosis due to an increased plasma concentration of Exp‐3174, which is more potent than LOS.

Increasing evidence suggests that autophagy, an evolutionarily conserved mechanism which eliminates dysfunctional organelles and long‐lived proteins, plays critical roles in maintaining normal cardiac function 47. In particular, mitophagy is an essential organelle‐specific autophagy in the heart, because the heart is one of the most stressed organs and is, thus, susceptible to mitochondrial damage 28. Based on the results of our current study, we were able to corroborate the previous finding that mitophagy is upregulated after transient activation of general autophagy ceases, thereby playing a protective role in alleviating LV dysfunction in response to PO 29. In addition, consistent with the fact that administration of SIM activates mitophagy 10, we validated that SIM enhances mitophagic activity during PO in mouse hearts. However, the mitophagy machinery activated by SIM administration is attenuated by the presence of LOS in the mouse hearts subjected to PO. By considering this and the fact that the cardioprotective effects of LOS + SIM are significantly greater than those of SIM in the absence of AT1R, we speculate that the beneficial effect of co‐administration of LOS and SIM during PO is mediated through AT1R negative signaling induced by inverse agonists, including Exp‐3174. Although we could speculate that AT1R‐mediated negative signaling by treatment with AT1R inverse agonists, such as Exp‐3174, is critical for the SIM‐mediated activation of mitophagy, conducting future experiments to verify this hypothesis is necessary.

There are several limitations of this study. First, we did not consider other underlying mechanisms of salutary effects of combined LOS and SIM against heart failure, such as anti‐inflammatory activities. Second, as there are several other versions of statins and ARBs, it will be necessary to determine whether other combinations of statins and ARBs are able to alleviate heart failure. Moreover, the results obtained here should be verified in large‐scale clinical studies.

## Conclusion

The present study demonstrates that combination LOS and SIM therapy has beneficial synergistic effects in animal models of heart failure. Such an approach may provide a novel therapeutic option in the treatment of human patients with this intractable disease.

## Author contributions

YM and YI planned the experiments, performed the experiments, analyzed the data, and wrote the manuscript. NT, YS, MK, TA, KH, and MI interpreted the experiments and analyzed the data. YM planned the experiments, analyzed data, modified the paper, and approved the final version of the manuscript submitted for publication.

## Conflict of interest

This study was supported by MERCK & Co., Inc. by providing a grant and compounds. The sponsor of the study had no role in the study design, conduct of the study, data collection, data interpretation, or preparation of the report.
